# Comparison of the 2021 International Society for Stem Cell Research (ISSCR) guidelines for “laboratory-based human stem cell research, embryo research, and related research activities” and the corresponding Japanese regulations

**DOI:** 10.1016/j.reth.2022.05.002

**Published:** 2022-06-03

**Authors:** Hideki Yui, Kaori Muto, Yoshimi Yashiro, Saori Watanabe, Yukitaka Kiya, Ayako Kamisato, Yusuke Inoue, Zentaro Yamagata

**Affiliations:** a University of Yamanashi, Japan; b University of Tokyo, Japan; c Tokyo Metropolitan Geriatric Medical Center, Japan

**Keywords:** Ethical guidelines, Human stem cells, Human embryos, International society for stem cell research, Japan, ESCs, embryonic stem cells, iPSCs, induced pluripotent stem cells, ISSCR, International Society for Stem Cell Research

## Abstract

This paper presents a comparison of the 2021 guidelines for stem cell research and clinical translation outlined by the International Society for Stem Cell Research (ISSCR) with the current regulations in Japan regarding the performance of such research. This paper provides a convenient English-language summary of the Japanese regulations, and illustrates the difference between the ISSCR guidelines and Japanese regulations regarding the conditions of implementation of study activities using human embryos or stem cells, for researchers outside Japan. The regulations governing the performance of research activities using human embryos or stem cells in Japan are relatively complex and comprise a range of laws and guidelines; the specific rules applied depend on the characteristics of each study. Therefore, even similar research activities may differ in terms of not only the guidelines or laws implemented, but also the procedures required. Such situations may confuse researchers.

## Highlights

• This paper compares the 2021 guidelines of the ISSCRwith the current regulations of Japan.
• The ISSCR guidelines and Japanese regulations differ in several respects.
• The complex regulations in Japan may confuse researchers.

## 1 Introduction

In May 2021, the International Society for Stem Cell Research (ISSCR) released the latest edition of its guidelines for stem cell research and clinical translation [1]. In this update version of its guidelines, the ISSCR withdrew its endorsement of the so-called “14-day rule,” which is an international standard, enshrined in the law of several countries, that a human embryo should not be cultured outside the body after 14 days, as this is the typical threshold for the appearance of the primitive streak. This change in the ISSCR's view of the rule has attracted a great deal of attention, and was even reported in the Japanese news media [2]. Notably, the guidelines also include new regulations regarding human embryo and stem cell-derived embryo models, chimeras, organoids, and genome editing [3].

The authors of the present study have previously compared the ISSCR guidelines with the corresponding rules established by the Japanese government for Japanese researchers, and published the results in a Japanese paper [4]. The present paper is targeted at researchers in countries other than Japan. Therefore, this paper provides a convenient English-language summary of the contents of the aforementioned paper. In addition, as original content, this paper provides a simple description of the ethical regulations pertaining to medical research conducted in Japan (Section 2-1), describes the difference between the ISSCR guidelines and Japanese regulations regarding the conditions of implementation of study activities using human embryos or stem cells, and the problems faced by researchers in carrying out their research activities with specific examples (Section 4).

## 2 The ethical regulations that pertain to medical research in Japan

In Japan, specific procedures for ethical application of medical research are stipulated in relevant laws and government administrative guidelines. Table 1 lists some laws and guidelines pertaining to medical research involving human participants that were in effect in Japan as of November 2021.

**Table 1:** The laws and guidelines pertaining to medical research involving human participants that came in effect in Japan as of November 2021.

The law or guidelines	Year of establishment (the latest revision)

(1) Act on Securing the Quality, Efficacy, and Safety of Products, Including Pharmaceuticals and Medical Devices	1960 (2019)
(2) Act on the Regulation of Human Cloning Techniques (Note: The detailed regulations for some of the research activities referred to in this paper are provided in the Guidelines for the Handling of Specified Embryos, which was established under this law in 2001 and last revised in 2021.)	2000 (2014)
(3) Act on the Safety of Regenerative Medicine	2013 (2018)
(4) The Clinical Trials Act	2017 (2019)
(5) Ethical Guidelines for Medical and Biological Research Involving Human Subjects	2021 (2022)
(6) Guidelines for Gene Therapy Clinical Trial	2015 (2022)
(7) Guidelines on the Derivation of Human ES Cells	2014 (2022)
(8) Guidelines on the Utilization of Human ES Cells	2019 (2022)
(9) Guidelines for the Distributing Institute of Human ES Cells	2019 (2022)
(Note. The first guidelines for ESCs were established in 2001 and reorganized several times after that.)	
(10) Ethical Guidelines for Assisted Reproductive Technology Research that Involves the Generation of Human Embryos	2010 (2022)
(11) Guidelines on the Research on Producing Germ Cells from Human iPS Cells or Human Tissue Stem Cells	2010 (2022)
(12) Ethical Guidelines for Research that Involves the Use of Technology to Modify Genetic Information in Human Embryos	2019 (2022)

In the cases of laboratory-based general medical research involving human participants, the Ethical Guidelines for Medical and Biological Research Involving Human Subjects stipulate that relevant studies should be conducted only after an ethical review has been approved by the research ethics committees defined by the guidelines. (In this paper, “should be conducted” is used for cases where the guidelines or laws clearly define the conditions of implementation of the study activities or allow the study activities. “Can be conducted” is used in the cases where the guidelines or laws do not define the conditions and though researchers may be confused, in fact they are allowed to conduct relevant studies. “Could be conducted” is used in the cases where the guidelines or laws do not prohibit clearly, but it appears that researchers are not allowed to conduct research activities in fact.) However, in cases of research involving “materials and data for which the academic value has already been established, that are widely used in research, and that can generally be obtained” (Article 3), which do not involve intervention in the human body, the guidelines do not apply and an ethical review is not necessary. For example, in-vitro research that uses cell lines without any clinical information obtained from a biobank such as Riken BRC Cell Bank in Japan, is included in this category. Although, specialized studies, such as those that utilize human-animal chimeric embryos, human ESCs, gene editing to the human embryos, gamete genesis from human iPSCs creating new human embryo are subject to specific laws or guidelines, and must undergo a specialized ethical review that accords with the applicable regulations. In order to clarify the problems of this situation in Japan, where there are several similar guidelines, we will compare the ISSCR guidelines with the Japanese regulations.

## 3 Comparison of the 2021 edition of the ISSCR guidelines and corresponding Japanese regulations

### 3.1 Contents of the 2021 edition of the ISSCR guidelines

The contents of the 2021 edition of the ISSCR guidelines are as follows:

1. Fundamental Ethical principles
2. Laboratory-based Human Embryonic Stem Cell Research, Embryo Research, and Related Research Activities
3. Clinical translation of stem cell-based interventions
4. Communications
5. Standards in stem cell research

The main focus of this paper is “2. Laboratory-based Human Embryonic Stem Cell Research, Embryo Research, and Related Research Activities.” This section defines the “specialized scientific and ethics oversight process” and illustrates what kind of research activities using human embryos or stem cells should be permitted and be reviewed by such oversight processes. Therefore, we can understand the ISSCR's attitude toward various laboratory-based research activities using human embryos or stem cells in this section. Thus, this section is suitable for comparison with the situation in Japan, wherein separate guidelines and laws have been established for similar research activities.

The “specialized scientific and ethics oversight process” refers to committees, other than the research ethics committees that conduct examinations of general medical studies, and examine studies involving specified stem cells and embryos. In the context of Japan, it would seem that the specialized reviews stipulated in the various guidelines or laws for research on ESCs and embryos mentioned in the following section correspond to this.

Regarding the issue of which types of studies involving stem cells and embryos are allowed after examination by a specialized scientific and ethics oversight process, the ISSCR guidelines feature the following five categories: 1A (Exempt from review by a specialized oversight process), 1B (Reportable, but not typically reviewed by a specialized oversight process), 2 (Reviewed by a specialized oversight process), 3A (Not allowed: currently unsafe), and 3B (Not allowed: lacks compelling scientific rationale or is ethically concerning).

The following sections describe the research activities regulated by each category and the corresponding regulations in Japan.

### 3.2 Category 1A: exempt from review by a specialized oversight process

According to the guidelines, this category “relates to research activities that are determined to be exempt from a specialized scientific and ethics oversight process after being assessed by the appropriate existing mandates and committees for laboratory research.” The Japanese regulations for such research activities are presented in Table 2, alongside the ISSCR guidelines for this category.

**Table 2:** Research allocated under category 1A in the ISSCR guidelines and the corresponding regulations in Japan.

ISSCR Guidelines – Research activities that are exempt from review by a specialized oversight process	The Japanese Regulations
a. Research with human pluripotent stem cell lines that is confined to cell culture and involves routine research practices, such as differentiation into tissue-specific cell types.	In cases in which ESCs are not used, such research should be conducted in accordance with the Ethical Guidelines for Medical and Biological Research Involving Human Subjects. In cases in which ESCs are used, such research should be conducted in accordance with the Guidelines on the Utilization of Human ES Cells.
b. Research that entails the reprogramming of human somatic cells to pluripotency (for example, the generation of induced pluripotent stem cells).	Such research should be conducted in accordance with the Ethical Guidelines for Medical and Biological Research Involving Human Subjects.
c. Research that entails the use of human fetal tissue and cells.	Such research can be conducted in accordance with the Ethical Guidelines for Medical and Biological Research Involving Human Subjects.
d. Research on stem cell culture systems that model specific stages of development or specific anatomic structures rather than the continuous development of an intact embryo or fetus.	In cases in which ESCs are not used, such research should be conducted in accordance with the Ethical Guidelines for Medical and Biological Research Involving Human Subjects. In cases in which ESCs are used, such research should be conducted in accordance with the Guidelines on the Utilization of Human ES Cells.
e. The transplantation of human stem cells, their derivatives, or other human cells into postnatal animal hosts.	

### 3.3 Category 1B: reportable, but not typically reviewed by a specialized oversight process

According to the guidelines, this category comprises “research that is reportable to the entity or body responsible for the specialized scientific and ethics oversight process, but not normally subject to further or ongoing review, at the discretion of the entity responsible for the oversight process and subject to regulations and policies in the jurisdiction.” The corresponding regulations in the Japanese system are illustrated in Table 3.

**Table 3:** Research allocated to category 1B in the ISSCR guidelines and the corresponding regulations in Japan.

ISSCR Guidelines – Research activities that are reportable, but not typically reviewed by a specialized oversight process	The Japanese Regulations
a. Research that entails the *in vitro* formation of human stem cell-based embryo models that are not intended to represent the integrated development of the entire embryo including its extraembryonic membranes.	In cases in which ESCs are not used, such research should be conducted in accordance with the Ethical Guidelines for Medical and Biological Research Involving Human Subjects. In cases in which ESCs are used, such research should be conducted in accordance with the Guidelines on the Utilization of Human ES Cells.
b. Chimeric embryo research in which human pluripotent stem cells are transferred into non- human, mammalian embryos and cultured *in vitro* for the minimum time necessary to achieve the scientific objective without gestation.	Such research should be conducted in accordance with the Act on the Regulation of Human Cloning Techniques.
c. Research on *in vitro* gametogenesis from human cells including genetically modified pluripotent stem cells, which does not involve attempts at fertilization and the generation of embryos.	In cases of gametogenesis from iPSCs, such research should be conducted in accordance with the Guidelines on the Research on Producing Germ Cells from Human iPS Cells or Human Tissue Stem Cells. In cases of gametogenesis from ESCs, such research should be conducted in accordance with the Guidelines on the Utilization of Human ES Cells.

### 3.4 Category 2: reviewed by a specialized oversight process

The corresponding Japanese rules for studies of this category are shown in Table 4. Supplementary information pertaining to such studies is outlined below.

**Table 4:** Research that is allocated to category 2 of the ISSCR guidelines and the corresponding regulations in the Japanese system.

ISSCR Guidelines - Research activities that should be reviewed by a specialized oversight process	The Japanese Regulations
a. Procurement and use of IVF human embryos for research *in vitro*.	The generation of ESCs, the replacement of mitochondria in embryos, and the genetic alteration in embryos, should be conducted in accordance with the corresponding set of guidelines (Guidelines on the Derivation of Human ES Cells, Guidelines for the Handling of Specified Embryos, and Ethical Guidelines for Research that Involves the Use of Technology to Modify Genetic Information in Human Embryos, respectively). For uses other than those listed above, such research can be conducted in accordance with the Ethical Guidelines for Medical and Biological Research Involving Human Subjects.
b. Procurement of human gametes to create research embryos *in vitro*.	Such research should be conducted in accordance with the Ethical Guidelines for Assisted Reproductive Technology Research that Involves the Generation of Human Embryos.
c. Research that generates human gametes from any progenitor cell type *in vitro*, when this entails performing studies of fertilization that produce human zygotes and embryos. The gametes may be derived from human pluripotent stem cells, oogonia, or spermatogonial stem cells that have been maintained *in vitro*, and they may be genetically modified or not.	Such research is prohibited by the Guidelines on the Research on Producing Germ Cells from Human iPS Cells or Human Tissue Stem Cells, as well as the Guidelines on the Utilization of Human ES Cells.
d. Research involving the genetic alteration of human embryos or gametes used to make embryos *in vitro*.	The genetic alteration of surplus embryos should be conducted in accordance with the Ethical Guidelines for Research that Involves the Use of Technology to Modify Genetic Information in Human Embryos. The generation of embryos by genetic altered germ cells, and the genetic alteration of new embryos should be conducted in accordance with the Ethical Guidelines for Assisted Reproductive Technology Research that Involves the Generation of Human Embryos.
e. Derivation of new cell lines from human embryos (not confined to pluripotent cell lines).	Regarding ESCs, research should be conducted in accordance with the Guidelines on the Derivation of Human ES Cells. In cases in which surplus embryos are used to derivate cell lines other than Human ESCs, such research can be conducted in accordance with the Ethical Guidelines for Medical and Biological Research Involving Human Subjects. In cases in which the research activity uses new embryos to derivate cell lines other than Human ESCs and is determined to be within the scope of the research objectives stipulated in the Ethical Guidelines for Assisted Reproductive Technology Research that Involves the Generation of Human Embryos, such research can be conducted in accordance with these ethical guidelines.
f. Research involving the *in vitro* culture of human embryos where embryos are maintained in culture until the formation of the primitive streak or 14 days from fertilization, whichever occurs first.	The cultivation of human embryos up to 14 days from fertilization is allowed, provided the procedure is in accordance with the following guidelines: For cases in which new embryos are generated: Ethical Guidelines for Assisted Reproductive Technology Research that Involves the Generation of Human Embryos. For cases of the generation of ESCs using surplus embryos: Guidelines on the Derivation of Human ES Cells. For cases of the replacement of the mitochondria of surplus embryos: Act on the Regulation of Human Cloning Techniques. The genetic alteration of surplus embryos: Ethical Guidelines for Research that Involves the Use of Technology to Modify Genetic Information in Human Embryos. For the use of surplus embryos, other than those listed above, there are no specific regulations. Thus, such research activity can be conducted in accordance with the Ethical Guidelines for Medical and Biological Research Involving Human Subjects.
g. Generation of stem cell-based embryo models that represent the integrated development of the entire embryo including its extraembryonic membranes.	In cases in which ESCs are not used, such research can be conducted in accordance with the Ethical Guidelines for Medical and Biological Research Involving Human Subjects. In cases in which ESCs are used, such research can be conducted in accordance with the Guidelines on the Utilization of Human ES Cells.
h. Research aimed at generating human totipotent cells that have the potential to sustain embryonic development *in vitro*.	To generate totipotent cells, it is necessary to develop embryos. Therefore, if such research is determined to be within the scope of the research objectives stipulated in the Ethical Guidelines for Assisted Reproductive Technology Research that Involves the Generation of Human Embryos, it can be conducted in accordance with these ethical guidelines using new embryos. In cases in which surplus embryos are used, such research can be conducted in accordance with the Ethical Guidelines for Medical and Biological Research Involving Human Subjects.
i. Chimera research in which human pluripotent stem cells or their derivatives with broad potential are introduced into: a) a non-human embryo or fetus *in utero*; or, b) a non-human embryo *in vitro* followed by transfer into a non-human uterus. Such experiments—if they are scientifically justified for the use of non-human primates above all other laboratory species—must exclude great and lesser ape species hosts (i.e., chimpanzees, gorillas, orangutans, bonobos, gibbons, and siamangs), as apes are prohibited from being used for invasive research in most parts of the world.	If the research corresponds to condition a) and does not involve the use of ESCs, it can be conducted in accordance with the Ethical Guidelines for Medical and Biological Research Involving Human Subjects. If such research does involve the use of ESCs, it can be conducted in accordance with the Guidelines on the Utilization of Human ES Cells. Research corresponding to condition b) should be conducted in accordance with the Act on the Regulation of Human Cloning Techniques.
j. Transferring human embryos to a human uterus following mitochondrial replacement.	Such research is prohibited by the Act on the Regulation of Human Cloning Techniques. *Note*. This act permits the replacement of mitochondria *in vitro* using surplus embryos.

When comparing the types of studies that the ISSCR guidelines require to be reviewed by a specialized oversight process with the corresponding Japanese regulations, the first notable point of concern is the handling of the so-called “surplus embryos,” which are frozen and donated from infertile couples. In Japan, research using new embryos is allowed under the Ethical Guidelines for Assisted Reproductive Technology Research that Involves the Generation of Human Embryos. In such cases, studies are limited to the purpose of developing assisted reproductive technology. Studies using new embryos, including those that involve the genetic alteration in new embryos and the generation of embryos that are fertilized using germ cells that are genetically altered, should be conducted under these guidelines. However, the regulations for the use of surplus embryos differs depending on the study type. There are specific guidelines or laws for the generation of ESCs, replacement of mitochondria in embryos, human-animal chimeric embryos, and gene editing in embryos. The use of surplus embryos is allowed for studies involving such activities with specialized review. However, the situation is unclear regarding the handling of surplus embryos for research activities such as embryological research or basic research for fertility treatments, other than those indicated above; hence, such studies can be conducted in accordance with the Ethical Guidelines for Medical and Biological Research Involving Human Subjects without specialized review.

The second notable contrast between the ISSCR guidelines for this category and the Japanese regulations is in the handling of apes. Research activities described in this paper utilizing apes (chimpanzees, gorillas, orangutans, bonobos, gibbons, and siamangs) are prohibited under the ISSCR guidelines. However, the Japanese guidelines pertaining to research ethics contain no regulations mandating the protection of apes. Nevertheless, as the capture, conveyance, sale, and export of endangered species are, as a general rule, prohibited by the Act on Conservation of Endangered Species of Wild Fauna and Flora, and as many apes are in danger of extinction, in reality it is extremely difficult to utilize apes in experiments.

While the culture of a human embryo within 14 days is classified under Category 2 (f), studies involving cultures of human embryos beyond this period do not appear in the lists of categories in ISSCR guidelines. Nevertheless, the guidelines recommend that if local policies and regulations permit, and with enough public support, the specialized oversight process may address such a study.

### 3.5 Category 3A: (not allowed: currently unsafe); category 3B: (not allowed: lacks compelling scientific rationale or is ethically concerning)

The research activities regulated under Category 3 of the ISSCR guidelines are illustrated below. Such studies are either not permitted under the Japanese regulations or are exceedingly difficult to conduct. Thus, we did not create a table indicating the corresponding regulations in the Japanese system for each study type. The specific Japanese laws and guidelines that prohibit these types of studies differ depending on the contents of the research activities.

3A
a Research in which human embryos that have undergone modification to their nuclear genome are transferred into or gestated in a human uterus.
b Research in which human embryos that have undergone editing to their mitochondrial genome are transferred into or gestated in a human uterus, as current knowledge of such interventions is inadequate to ensure safety.
c The use of human gametes differentiated from human stem cells for the purposes of fertilization and human reproduction.
3B
a Transfer of human stem cell-based embryo models to the uterus of either a human or animal host.
b Research in which human embryos produced by reprogramming of nuclei are implanted into a human or animal uterus (often referred to as human reproductive cloning).
c Research in which animal chimeras incorporating human cells with the potential to form human gametes are bred to each other.
d Transfer of chimeric embryos mixing animal and human cells (whether predominantly animal or human) to the uterus of a human or great or lesser ape (i.e., chimpanzees, gorillas, orangutans, bonobos, gibbons, and siamangs).
e Transfer of a human embryo(s), irrespective of its origins, to an animal uterus.

Other than the two exceptions, the above research activities are clearly prohibited by the Japanese system. In other words, there are no specific regulations outlined about these exceptions in the Japanese laws and guidelines. The first exception is 3B (a). In the cases of transfer of human embryo models into animal uteri, if this activity does not involve the utilization of ESCs, it could be conducted in accordance with the Ethical Guidelines for Medical and Biological Research Involving Human Subjects; if this activity involves the utilization of ESCs, it could be conducted in accordance with the Guidelines on the Utilization of Human ES Cells. Additionally, even though cell lines, except for ESCs, obtained from a biobank without any clinical information are utilized, and the Ethical Guidelines for Medical and Biological Research Involving Human Subjects are not applied, such research nevertheless needs to be reviewed by an animal experiments committee. In the case of the transfer of a human embryo model into human uteri, it could be conducted in accordance with the Act on the Safety of Regenerative Medicine. However, it is extremely unlikely that research of this type would be allowed under individual ethical reviews.

The second exception is a part of 3B (d): The transfer of chimeric embryos mixing animal and human cells to the uterus of a great or lesser ape. Japanese laws and guidelines concerning research ethics do not include regulations requiring the protection of apes. Therefore, despite the fact that it is considered extremely difficult to utilize apes—many of which are in danger of extinction—in experiments, consideration of the Act on the Regulation of Human Cloning Techniques indicates that the transfer of animal-human chimeric embryos into ape uteri is not prohibited. Nevertheless, it is extremely unlikely that such activity would be approved by the ethical review required by the law.

## 4 Discussion

We described the difference between the ISSCR guidelines and Japanese regulations regarding the conditions of implementation of study activities using human embryos or stem cells. The study types that require a specialized scientific and ethical oversight process according to the ISSCR guidelines but not according to the Japanese regulations, fall under some sections of Category 2 (a), (e), (f), (g), (h), and (i). The study types that do not require such process according to the ISSCR guidelines but do so according to the Japanese regulations fall under Category1-B(b), (c), and some sections of Category1-A(a), (d), (e), and Category1-B(a). The study types that are allowed under the ISSCR guidelines but are prohibited under the Japanese rules are Category2 (c), and (j).

Comparing the ISSCR guidelines with Japanese laws and guidelines for all categories, it is notable that there are no Japanese laws or guidelines that establish the general principles of procedures necessary for research activities using human embryos. Japanese regulations of research that use human embryos are complex. They require the research to be conducted in accordance with specific laws and guidelines depending on the contents with a review, which corresponds to the specialized scientific and ethical oversight process of the ISSCR guidelines. Even if the research activities are similar, there are subtle differences in the review processes and procedures. One instance is the genetic alteration of human embryos, which is defined under Category 2 (d) in the ISSCR guidelines. According to the Japanese regulations, the genetic alteration of surplus embryos should be conducted in accordance with the Ethical Guidelines for Research that Involves the Use of Technology to Modify Genetic Information in Human Embryos (the former), and the genetic alteration of new embryos should be conducted in accordance with the Ethical Guidelines for Assisted Reproductive Technology Research that Involves the Generation of Human Embryos (the latter). Both guidelines require researchers to undergo the review process of the research ethics committee in the research institution and confirm with the government that the research plan conforms to the guidelines. (Following this, the government confirms with the special committee whether the research plan conforms to the guidelines). However, the requirements for membership on the research ethics committee slightly different in both guidelines. The former requires the expert in reproductive medicine, the expert in genetic alteration technology, the expert in bioethics, the legal expert or other expert in the humanities and social sciences, and those who can advocate general public's opinion, while the latter requires additional expert in biology. In addition, although there are many commonalities in the format of documents to be submitted to the government defined by both guidelines, researchers must use different forms. [5,6]

Furthermore, research activities that use surplus embryos beyond the scope of specific laws and guidelines can be conducted in accordance with the Ethical Guidelines for Medical and Biological Research Involving Human Subjects. Thus, such activities can be conducted without a specialized ethical review, which corresponds to the specialized scientific and ethical oversight process of the ISSCR guidelines. However, specific laws and guidelines are established on the grounds that human embryos are “emerging potential of human life” and provide for specialized review. Such situation lacking logical coherence may confuse researchers. Therefore, it can be said that complexity and ambiguity are some of the problems of the Japanese regulations.

## 5 Conclusion

This paper presented a comparison of the 2021 edition of the ISSCR guidelines and the corresponding Japanese regulations. This afforded a visualization of the extremely complex Japanese system, which features a range of laws and guidelines for the conducting of medical research using human embryos or stem cells, with the applicable laws/guidelines depending on the specific contents of studies. In addition, this paper highlighted the difference between ISSCR guidelines and Japanese regulations regarding the conditions of implementation of study activities using human embryos or stem cells.

## Funding

This research was supported by 10.13039/100009619AMED, under grant number JP 21bm0904002.

## Declaration of competing interest

The authors have no conflicts of interest to declare.
